# Redox-constrained microbial ecology dictates nitrogen loss versus retention

**DOI:** 10.1093/ismeco/ycaf219

**Published:** 2025-11-25

**Authors:** Jemma Fadum, Xin Sun, Emily Zakem

**Affiliations:** Division of Biosphere Sciences and Engineering, Carnegie Institution for Science, Stanford, CA 94305, United States; Division of Biosphere Sciences and Engineering, Carnegie Institution for Science, Stanford, CA 94305, United States; Xin Sun, Department of Biology, University of Pennsylvania, Philadelphia, PA 19104, United States; Division of Biosphere Sciences and Engineering, Carnegie Institution for Science, Stanford, CA 94305, United States

**Keywords:** denitrification, DNRA, anammox, OMZ, anoxic environments, water column, nitrite reduction

## Abstract

Microorganisms drive biogeochemical cycling. Therefore, examining environmental change through the lens of microbial ecology is particularly useful for developing a mechanistic understanding of the biogeochemical consequences and feedbacks of perturbations to ecosystems. When aquatic systems with deep anoxic waters undergo eutrophication, the resulting surface productivity impacts the anaerobic microbial community below. The increase in sinking organic carbon can shift the anaerobic community function from inorganic nitrogen (N) loss to N retention, amplifying eutrophication as a positive feedback. However, we lack a mechanistic understanding of this transition, which is critical for anticipating these impacts in aquatic environments where microbial community composition is unknown. Here, we provide a first-principles, quantitative model of this transition from N loss to retention by linking ecological dynamics to the energetics underlying microbial metabolisms. We develop and analyze an ecosystem model in which redox chemistry constrains the traits of key anaerobic N-cycling microbial functional types: denitrification, dissimilatory nitrate reduction to ammonium, and anaerobic ammonium oxidation (anammox). The model captures the transition from N loss to N retention with increasing organic carbon supply, consistent with observations for specific systems and species. Results identify characteristics of the microbial community composition at the “net zero N loss” point at which N loss balances N retention, providing testable hypotheses for sequencing data and other observations. By tying microbial ecological dynamics to environmental chemical potential, results provide a broadly applicable framework for better predicting the biogeochemical impacts of eutrophication, deoxygenation, and other perturbations.

## Introduction

Aquatic ecosystems face a myriad of impacts related to global change, from anthropogenically driven changing climate patterns to more localized disturbances. Such impacts, whether local or global, often manifest through changes in microbial community function. One ubiquitous impact on both freshwater and coastal ecosystems is eutrophication, characterized broadly by increased algal productivity and accompanying ecosystem changes caused by excessive nutrient supplies from sources such as the overapplication of fertilizers, untreated (or undertreated) municipal waste, and net pen aquaculture. The algal blooms that develop in such systems can subsequently produce hypoxic (low oxygen) or anoxic (often undetectable oxygen) zones in the water column as decomposing algal biomass and other OM sinks below the ventilated surface waters [1]. Often called “dead zones” for their inability to support marine megafauna, these low oxygen environments are still teeming with microbial life [2].

When oxygen is unavailable, microbial communities operate anaerobically by using oxidized forms of inorganic nitrogen (N) and other molecules as electron acceptors. Therefore, the development of anoxic zones plays a critical role in both local and global N cycling dynamics. When oxygen supply is negligible, three anaerobic metabolisms (or else a subset of the three) can be expected to dominate anoxic water column N-cycling: denitrification (NO_3_^−^ ➔ NO_2_^−^ ➔ NO ➔ N_2_O ➔ N_2_), anaerobic ammonium oxidation (anammox; NH_4_^+^ + NO_2_^−^ ➔ N_2_), and dissimilatory nitrate reduction to ammonium (DNRA; NO_3_^−^ ➔ NO_2_^−^ ➔ NH_4_^+^).

The degree to which bioavailable N is lost (through denitrification or anammox) or retained (through DNRA) in a strictly anoxic aquatic ecosystem is thus primarily determined by the proportion of active denitrification, anammox, and DNRA. Denitrification and DNRA utilize similar types of reduced substrates as electron donors, such as OM if operating heterotrophically. Therefore, the populations carrying out these metabolisms can be considered to be competing for both NO_x_ and OM. In contrast, chemoautotrophic anammox is often understood to exist in syntrophy with denitrification, relying on the NH_4_^+^ excreted from the remineralization of OM by denitrifying heterotrophs [3–4].

**Figure 1:**
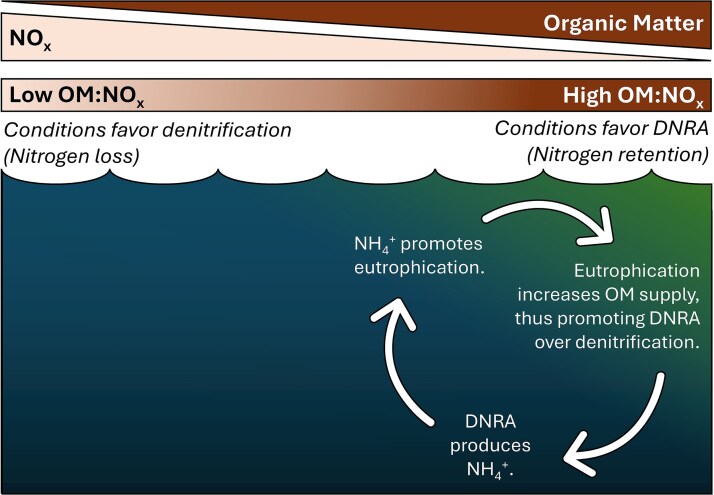
Conceptual diagram of the positive feedback where DNRA is promoted by eutrophication while simultaneously supplying nutrients which fuel eutrophication.

Previous research has demonstrated that, generally, DNRA is more favorable when microbial growth is NO_x_-limited whereas denitrification is more favorable when microbial growth is OM-limited [5–10], though exceptions to this pattern can occur in metabolically flexible organisms [11]. A transition to the dominance of DNRA (i.e. the N retention pathway) in increasingly OM-rich environments provides a positive feedback to eutrophication (Fig. 1). As eutrophication expands, elevated OM sinking from increasingly productive surface waters may transition the ecosystem from denitrification dominant to DNRA dominant, thus retaining bioavailable N and further spurring eutrophication by supplying inorganic N, which supports primary productivity. In the other direction, if supply of OM decreases, the system may shift towards a more denitrification dominant state, increasing bioavailable N loss, thus potentially reducing surface primary production and subsequently the supply of OM to anoxic zones below. This feedback is particularly important in aquatic ecosystems where primary productivity is N-limited (e.g. roughly half of the surface ocean and some inland bodies of water [12–13]).

Despite the ecological significance of this feedback loop, DNRA is often excluded from biogeochemical modeling efforts in both marine and freshwater environments ([14–16], among others). This exclusion in marine system models stems from observations that DNRA is an insignificant component of N cycling in global oxygen minimum zones. However, as coastal conditions and inland waters are becoming increasingly anoxic and eutrophic, the inclusion of DNRA in diverse modeling efforts is critical to making accurate projections of N cycling dynamics. Therefore, a mechanistic, quantitative understanding of the controls on these key metabolisms (denitrification, DNRA, and anammox) and, chiefly, the competition between N loss and retention pathways is required.

Jia *et al.* [10] provided one such interpretation of denitrification and DNRA competition using Resource Competition Theory [17], demonstrating that measured denitrification and DNRA outcomes can be predicted by the ratio of organic C to NO_3_^−^ supply. However, the model parameters were set by measured traits of specific microbial populations in culture, therefore masking the impact and diversity of the uncultivated majority [18]. Furthermore, given the taxonomic breath of both denitrification and DNRA-performing organisms [19], a more general model is needed in order to predict changes in unobserved, unculturable, and future-adapted communities.

One way forward is to link population traits to fundamental underlying constraints, foregoing species-specific descriptions in favor of more general and broadly applicable trait-based descriptions of functional types [20–21]. For metabolically diverse prokaryotes, the key redox reaction fueling a metabolism provides one such fundamental constraint [22–24]. In line with this approach, Algar and Vallino [8] optimized for maximum entropy production among N-cycling metabolisms in a coastal sediment environment, which, like Jia *et al.* [10], explained the niche differentiation of denitrification and DNRA according to the ratio of organic C to NO_3_^−^ supply. However, the study focused on sediments, rather than the water column, where the supply mechanisms and magnitudes of essential substrates differ. In addition to the fundamental differences between sediments and the water column environment, a model framework devised for the water column is needed to allow for integration with multi-dimensional circulation models of aquatic environments, including the biogeochemical models used for climate change projections.

**Figure 2:**
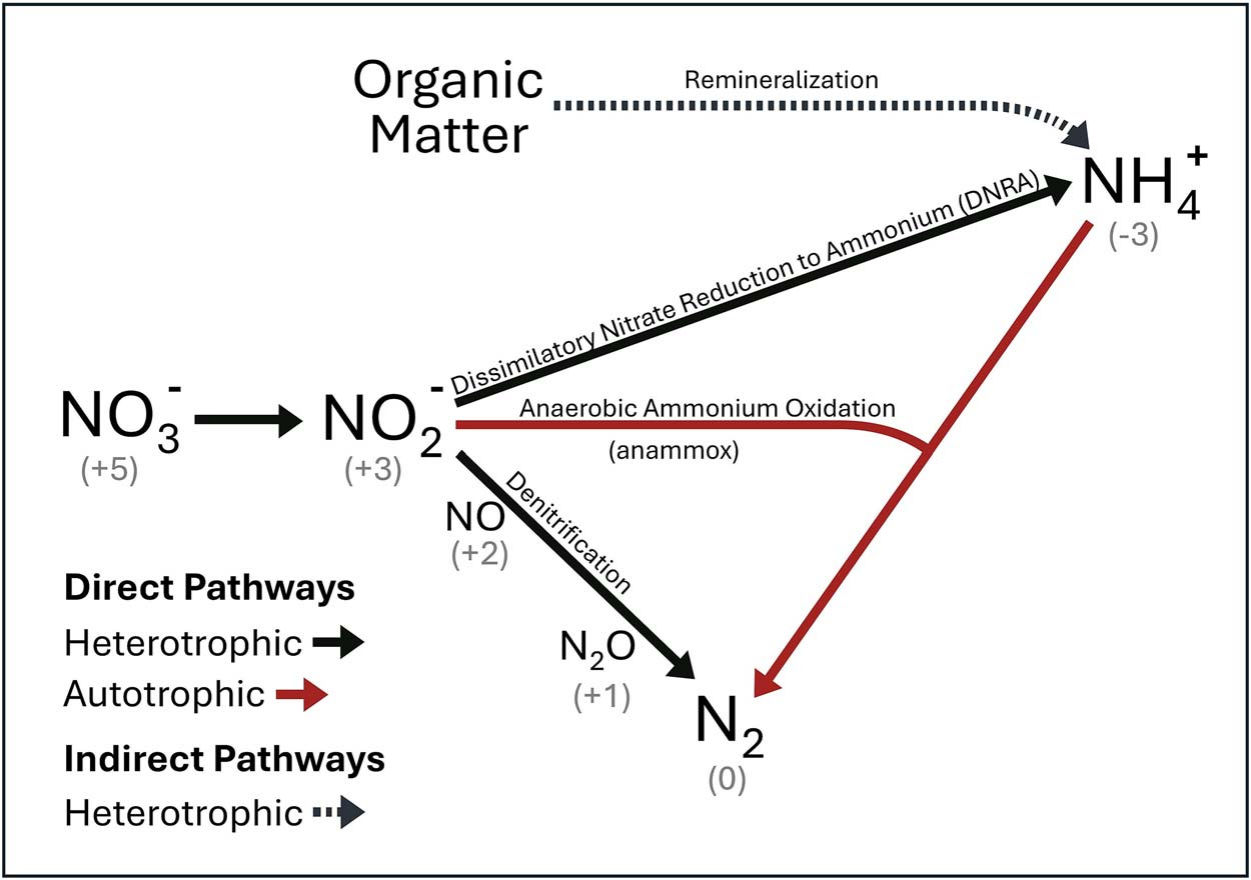
Conceptual diagram of the key anaerobic N-cycling pathways. The fate of nitrate (NO_2_^−^) is determined by three main metabolisms (denitrification, anammox, and DNRA), which are represented by microbial functional types in the ecosystem model.

In order to address this fundamental gap in our understanding of N loss and retention dynamics in diverse anoxic aquatic ecosystems (marine, inland waters, and even groundwater in confined aquifers), here we link environmental chemical potential (i.e. nutrient availability) to the ecological dynamics of microbial populations (functional types) carrying out key anaerobic N-cycling metabolisms (denitrification, DNRA, and anammox, Fig. 2). We develop and analyze an ecological model with microbial functional types that are parameterized with the underlying free energies and stoichiometries of the redox reactions that fuel growth [24], providing a theoretically grounded quantitative framework to study microbial N-cycling, in contrast to a species-specific approach. Like Jia *et al.* [10], we use Resource Competition Theory to understand the ecological outcomes. The tradeoff resulting in the niche differentiation of denitrification versus DNRA embedded in the underlying energetics emerges in the traits of the functional types, demonstrating the ability of the modeling strategy to link chemical potential to trait-based descriptions of microbial communities. We then analyze the transition between N loss and N retention from a microbial ecological perspective and identify the microbial community composition at the transition point. This provides testable hypotheses for sequencing datasets and other future observations and experiments.

## Materials and methods

### Redox-constrained microbial functional types

We employ an established framework that uses the underlying redox reactions fueling metabolisms to quantitatively describe the N-cycling microbial functional types responsible for the three main anaerobic N-cycling pathways: denitrification, DNRA, and anammox (Fig. 2; [25–26]). Specifically, we develop and analyze a heterotrophic DNRA functional type and add it to previously developed functional types for heterotrophic denitrification and chemoautotrophic anammox [26]. Briefly, substrate (OM and inorganic N) requirements for the heterotrophic functional types are quantified using the half reactions of oxidized and reduced substrates and an estimated proportion of electrons devoted to biomass synthesis versus respiration [23]. This reflects the free energies of the reactions with consideration of the departure from standard state due to typical concentrations of these solutes in anoxic waters. As in previous work [26], we align the magnitudes of the biomass yields for all heterotrophic functional types with a range of empirically estimated biomass yields (or, C use efficiency) on organic C for natural aquatic environments (0.2–0.3 mol biomass C per mol C [27]). Thus, the relative values of the yields reflect the underlying redox chemistry, while the absolute values reflect this empirically estimated baseline. See Sun *et al.* [26] for full, detailed methodology for determining yields. See Supplemental Text S1 for DNRA-specific details.

As the first step of DNRA and denitrification (NO_3_^−^ ➔ NO_2_^−^) is not distinguishable from a redox perspective, we focus on the competition for NO_2_^−^, the critical intermediate that determines the fate of bioavailable N [8, 28–30]. Due to the known modularity of denitrification [31], we resolve the denitrification pathway with two functional types that each carry out a single-step pathway (NO_2_^−^ ➔ N_2_O and N_2_O ➔ N_2_). We use a previously published parameterization for the chemoautotrophic anammox functional type that relies on measured yields [32–33]. We represent OM simply as one bulk pool with typical marine stoichiometry (C_6.6_H_10.9_O_2.6_N [34]), though the methodology is sufficiently flexible to consider different stoichiometries and specific substrates.

This results in the following metabolic budgets, with each normalized to the synthesis of N-based biomass (with stoichiometry C_5_H_7_O_2_N [23, 25]), here in simplified form (neglecting water and proton balancing) for conciseness. For heterotrophs, the budgets reflect the baseline biomass yield range of 0.2–0.3 mol biomass C per mol organic C, here written in terms of the midpoint (0.25 mol/mol), which is consistent with the empirically estimated 0.26 mol/mol for aquatic environments [27]:


$$\mathbf{DNRA}:\underset{\mathrm{OM}}{\underbrace{6.8\ {\mathrm{C}}_{6.6}\mathrm{N}}}+29.7\ {\mathrm{NO}}_2^{-}\to \underset{\mathrm{Biomass}}{\underbrace{{\mathrm{C}}_5\mathrm{N}}}+35.5\ {\mathrm{NH}}_4^{+}+39.9\ \mathrm{DIC} $$

$${\begin{align*} &\mathbf{Denitrification}:\underset{\mathrm{OM}}{\underbrace{4.2\ {\mathrm{C}}_{6.6}\mathrm{N}}}+50.6\ {\mathrm{N}\mathrm{O}}_2^{-}\to \underset{\mathrm{Biomass}}{\underbrace{{\mathrm{C}}_5\mathrm{N}}}+50.6\ {\mathrm{N}}_2\mathrm{O}+3.2\ {\mathrm{N}\mathrm{H}}_4^{+}+22.5\ \mathrm{DIC} \end{align*}}$$

$${\begin{align*} &\mathbf{Anammox}:80.7\ {\mathrm{N}\mathrm{O}}_2^{-}+70.4\ {\mathrm{N}\mathrm{H}}_4^{+}+5\ \mathrm{DIC}\to \underset{\mathrm{Biomass}}{\underbrace{{\mathrm{C}}_5\mathrm{N}}}+69.4\ {\mathrm{N}}_2+11.3{\mathrm{N}\mathrm{O}}_3^{-} \end{align*}}$$

### Ecosystem model

A virtual chemostat model is seeded with the anaerobic functional types (denitrification, DNRA, and anammox) and supplied with varying incoming concentrations of OM and NO_2_^−^ (Supplemental Table S1). Ammonium (NH_4_^+^) is also supplied to the chemostat at a rate that represents the NH_4_^+^ released by implied nitrate (NO_3_^−^) reduction (Supplemental Text S2). We numerically integrate the model to equilibrium for each combination of incoming concentrations.

In addition to the biomass yields (Table 1), the functional type descriptions require parameters dictating substrate uptake kinetics and population loss due to mortality. As in previous work [25–26], we have kept the substrate uptake kinetic parameters and loss rate parameters the same for all types (Supplemental Table S2) in order to examine the competitive outcomes produced by differences in the yields, which reflect the differences in the underlying redox chemistry. Specifically, the growth rate *μ* (d^−1^) for each functional type *i* on required resource *j* (*R_j_*) is calculated using Leibig’s law of the minimum as:


(1) \begin{equation*} {\mu}_i=\underset{j}{\min}\left({y}_{ij}{V}_j^{max}\frac{R_j}{R_j+{K}_j}\right) \end{equation*}

so that the maximum growth rate (*μ_max_*) is the product of the yield and the maximum uptake rate *V^max^* (d^−1^), where *V^max^* is the same for each functional type. The loss rates are set by the chemostat dilution rate. However, because of the additional uncertainty in comparing heterotrophic and chemoautotrophic metabolisms, we later forego this assumption for anammox and instead consider that it may have optimized its cellular machinery to achieve a different competitive outcome.

**Table 1:** Comparison of OM yield (*y*_OM_; mols biomass N per mol OM) and NO_2_^−^ yield (*y*_N_; mols biomass N per mol NO_2_^−^).

Metabolism	*y*_OM_	*y*_N_	OM^*^	NO_2_^−*^
DNRA	0.11–0.18	0.026–0.042	0.080–0.12	0.0032–0.0054
Denitrification (NO_2_^−^ → N_2_O)	0.19–0.29	0.015–0.024	0.048–0.074	0.0058–0.0097
Anammox	*NA*	0.0133	*NA*	0.0123

The range of DNRA and denitrification yields and organic matter and nitrite subsistence concentrations (OM^*^ and NO_2_^*^, respectively) reflects the range in the baseline biomass yield with respect to organic N (0.2–0.3 mols of biomass per mol N in OM oxidized). Anammox *y_N_* (and resulting NO_2_^−*^) from Lotti *et al.* [32].

Finally, we use Resource Competition Theory to interpret the results [17]. In the resulting resource ratio diagrams, the consumption vectors (CVs) are the ratios of the resource yields (i.e. *y*_N_/ *y*_OM_), representing the ratio of OM to NO_2_^−^ consumption for the heterotrophs.

## Results

### Dissimilatory nitrate reduction to ammonium-denitrification tradeoff reflects redox chemistry

In a steady-state ecosystem, where microbial growth is faster than changes in the environment, the competitive ability of each functional type for a nutrient is set by its resource subsistence concentration (R^*^), which is the minimum concentration that can sustain a population in the environment, and the concentration to which the population will deplete that resource when its growth is limited by that resource [17]. In our model, each subsistence concentration is set by the population’s specific loss rate (i.e. the chemostat dilution rate), uptake kinetics (here, the maximum uptake rate and the half saturation constant, Supplemental Table S2), and the biomass yield ([25–26], Table 1). For the competing heterotrophs, the subsistence concentrations for NO_2_^−^ (NO_2_^*^) and OM (OM^*^) differ solely because of the differences in the redox-informed biomass yields (Table 1). The biomass yields are calculated from the metabolic budgets ([25–26], Supplemental Text S1) and are inversely related to the subsistence concentrations (Table 1). A larger yield/smaller R^*^ for a nutrient means a given functional type is more competitive when that nutrient is limiting.

Results show that, with respect to the consumption of NO_2_^−^, the denitrifying functional type has a greater OM yield (*y*_OM_) than DNRA (Table 1), which is consistent with empirical observations of denitrification dominance under OM limiting conditions. Conversely, DNRA has a greater NO_2_^−^ yield (*y*_N_) than denitrification, which is consistent with DNRA’s observed dominance under NO_x_ limited conditions. Conversely, DNRA has the lowest NO_2_^−^ yield (~18% lower than denitrification), reflecting the slow growth and high energetic cost of chemoautotrophy [35].

These yield estimates demonstrate that the metabolic tradeoff between OM and N utilization embedded in the redox reactions of denitrification and DNRA emerges in the theoretically modeled traits, without a dependency on other physiological or ecological factors. This is consistent with our hypothesis that fundamental constraints underlying metabolisms (i.e. chemical potential energy) predominantly govern the fitness of populations carrying out metabolisms that are sufficiently comparable (i.e. heterotrophic denitrification versus DNRA, considering that both gain energy from the oxidation of the same OM). Differences in kinetic parameters, such as a higher affinity for NO_2_^−^ for DNRA, are likely to evolve in accordance with these metabolic niches (as reflected in the measured traits in Jia *et al.* [10]), but we hypothesize that traits such as uptake affinity reflect a cellular-level optimization and thus are unlikely to “override” the fundamental redox-level constraints for comparable metabolisms (i.e. heterotrophs). We next examine the outcomes of the interactions of these functional types in the ecosystem model.

### Ecological outcomes

We incorporate the denitrification, DNRA, and anammox functional types into a simplified ecosystem model in order to: (i) illustrate how the redox-based tradeoff between higher competitive ability for OM versus NO_2_^−^, for denitrification and DNRA, respectively, shapes community composition across variable resource availability, (ii) examine the metabolic niche for anammox, and (iii) examine how the ecological outcomes relate to the biogeochemical outcome: the balance between N retention versus N loss.

**Figure 3:**
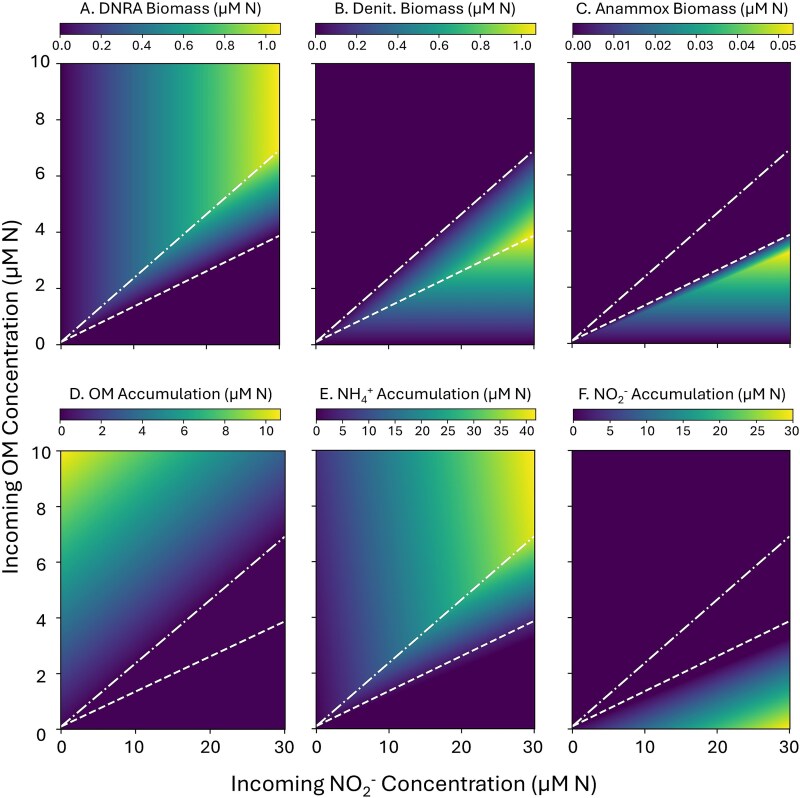
Multiple steady-state solutions of the ecosystem model with anaerobic N-cycling metabolic functional types across varying supply of OM and NO_2_^−^, showing biomass of (A) DNRA, (B) denitrification (NO_2_^−^ ➔ N_2_O + N_2_O ➔ N_2_), and (C) anammox. Chemostat accumulation (steady-state concentrations) of D) OM, E) NH_4_^+^ with F) NO_2_^−^. The white lines indicate the CVs for DNRA (− • − • −) and denitrification (---).

**Figure 4:**
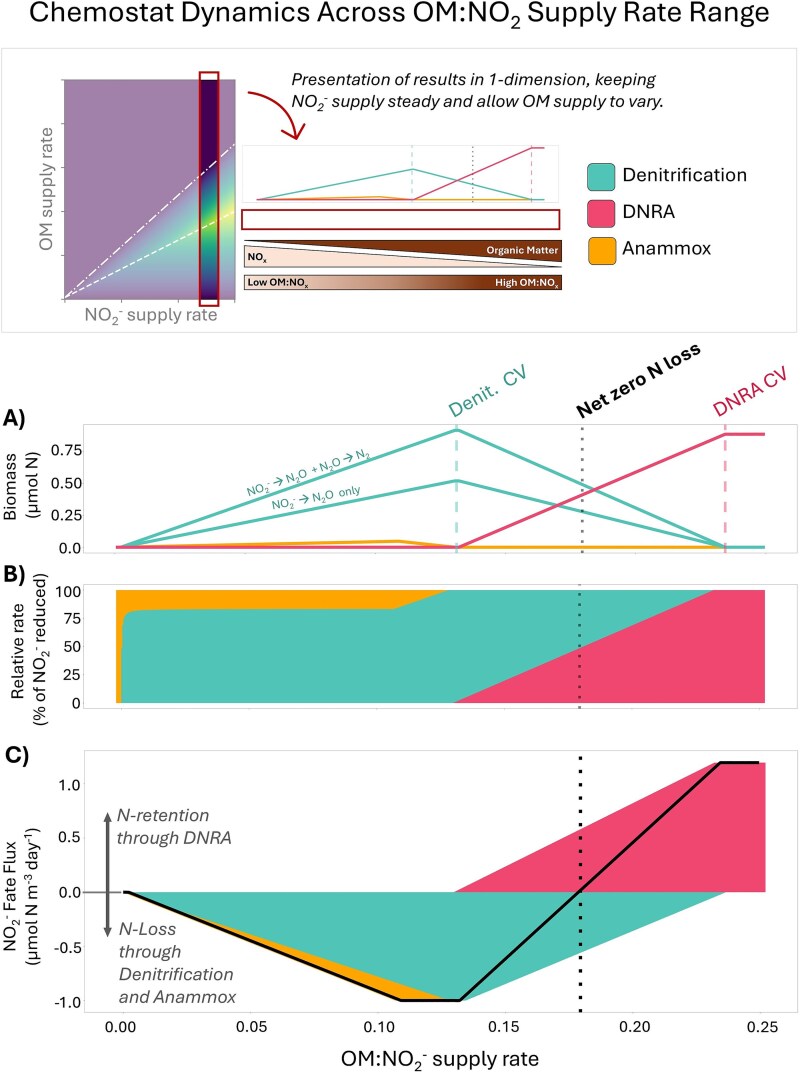
Ecological and biogeochemical outcomes governing the fate of NO_2_^−^ in the ecosystem model as a function of the relative supply of OM to NO_2_^−^ (here specifically with incoming NO_2_^−^ concentration of 25 *μ*M N and variable OM supply rate). At the top of the figure, an infographic demonstrates the relationship between these results and the steady state solutions presented in two-dimensional space in Fig. 3. (A) Biomasses of DNRA, denitrification, and anammox functional types, (B) percentage of NO_2_^−^ reduced via the three functional types, (C) N_2_ and NH_4_^+^ production, identifying point of net zero N loss.

Resulting steady-state model simulations show the variation in the ratio of active denitrification to DNRA as a function of OM:NO_2_^−^ supply rates (Fig. 3). To more clearly and quantitatively compare the biomasses, rates, and geochemistry, a subset of these simulations is illustrated in one dimension (a single NO_2_^−^ supply rate with varied OM supply rates) (Fig. 4). In low OM:NO_2_^−^ space, denitrification competitively excludes DNRA, and in high OM:NO_2_^−^ space, DNRA competitively excludes denitrification. These zones of exclusion are set by CV, which represent the relative consumption rates of OM versus N by the functional types. DNRA competitively excludes denitrification at OM:NO_2_^−^ supply rates above its CV, which is equal to the ratio of its biomass yields for OM and N (Fig. 3A and B). In contrast, denitrification competitively excludes DNRA at OM:NO_2_^−^ supply rates below its CV. Stable coexistence of denitrification and DNRA, despite their competition for both substrates, occurs where the two functional types are co-limited by both OM and NO_2_^−^, between the two CVs (Figs 3A and B, Fig. 4A).

These results are consistent with those of Jia *et al.* [10] yet without reliance on species-specific, measured parameters. Thus, the results of Jia *et al.* [10] serve as a test of the ability of our framework to successfully connect fundamental constraints (underlying energetics) to observed microbial communities, thus supporting the utility of this framework for anticipating dynamics in unobserved microbial communities where the species composition is unknown.

These competitive outcomes align with the patterns of nutrient accumulation. We consider a nutrient “accumulating” when its concentration is above the subsistence concentration of any microbial functional type. In low OM:NO_2_^−^ space, NO_2_^−^ (the electron acceptor) accumulates due to insufficient OM (the electron donor) to make use of the entire electron acceptor pool, Conversely, in high OM:NO_2_^−^ space, OM accumulates due to insufficient NO_2_^−^ to make use of the entire electron donor pool. In the coexistence regime, neither OM nor NO_2_^−^ accumulates because both limit microbial growth (Fig. 3D and F).

### The role of anammox

In the default version of the model, anammox is less competitive for NO_2_^−^ than the heterotrophs because of its very low NO_2_^−^ yield (*y_N_*) (Table 1). The resulting anammox biomass is generally an order of magnitude lower than both DNRA and denitrification biomass (Figs 3A–C and 4A), consistent with the observed low growth rate and low biomass accumulation in some natural environments [36]. Anammox is only sustained in the OM-limited regime at low OM:NO_2_^−^ supply rates, where there is sufficient NO_2_^−^ as well as sufficient NH_4_^+^ to serve as the electron donor. In this regime, anammox and denitrification maintain stable coexistence due to syntrophy, with the heterotrophs supplying NH_4_^+^ from the remineralization of the OM (Figs 3B, C and4A). In the default model, anammox is responsible for ~20% of NO_2_^−^ uptake (Fig. 4B) in the chemostat and ~26% of N_2_ production (Supplemental Fig. S1), which is consistent with the established geochemical calculations of the contribution of anammox to N loss in the ocean (~28%) reflecting control by the OM stoichiometry [3–4]. At higher OM:NO_2_^−^ supply, once NO_2_^−^ becomes depleted, anammox transitions from a state of NH_4_^+^ limitation to NO_2_^−^ limitation. Because the *y*_N_ of anammox is too low (and thus NO_2_^−*^ is too high) to compete with DNRA for NO_2_^−^, the chemoautotroph is eventually excluded as NO_2_^−^ becomes limiting.

However, given some observations of anammox co-occurring with DNRA in the ocean [37], we also considered the possibility that anammox may be a better competitor for NO_2_^−^ than the heterotrophs. Though we hypothesize from an evolutionary perspective that redox constraints should inform ecological niches of comparable metabolisms, such as heterotrophic denitrification and DNRA fueled by the same electron donor, our framework involves more uncertainty for comparing the niches of heterotrophs versus chemoautotrophs. Despite the low *y*_N_ of anammox in the default model, which reflects the high energetic cost of chemoautotrophy, it is plausible that anammox is able to devote more of its cellular machinery to NO_2_^−^ acquisition than the heterotrophs because it does not need to process complex organic substrates. If this cellular-level optimization is sufficient for anammox to have a lower NO_2_^−*^ than DNRA and denitrification (Supplemental Figs S2–S4), then the anammox functional type would be a superior competitor for NO_2_^−^ (despite observations and previous work suggesting otherwise [9, 32]). We conduct this model experiment and find that if this is the case, anammox can access NO_2_^−^ throughout the model domain and coexist with DNRA, with DNRA supplying NH_4_^+^ throughout the N-limited regime (Supplemental Figs S2 and S3).

Therefore, in this scenario where anammox is the better competitor for NO_2_^−^, (NO_2_^*^_anammox_ < NO_2_^*^_DNRA_ < NO_2_^*^_denitrification_), the model produces a domain of stable coexistence of all three functional types (Supplemental Figs S2–S3). This simulates the observed co-occurrence of all three functional types [28], although other mechanisms could also allow for this coexistence (see discussion). Below, we introduce the concept of net zero N loss, the point at which N retention balances loss, and we find that in this alternative model scenario, the stable coexistence of all three functional types results in a point of net zero N loss which occurs at a lower OM:NO_2_^−^ supply rate ratio than in the default model. However, qualitatively, we still see this transition occur at the halfway point between maximum denitrification and maximum DNRA biomass. Overall, this analysis generates a targeted question for observations and experiments about the competition between anammox and heterotrophs for NO_2_^−^.

### The balance between nitrogen loss and nitrogen retention

The outcome of the ecological interactions of the anaerobic N-cycling functional types results in varied fate of NO_2_^−^ across the resource ratio space. In high OM:NO_2_^−^ space, NH_4_^+^ production from DNRA dominates, and N is retained in the system (Fig. 4C). In low OM:NO_2_^−^ space, N_2_ production from denitrification and anammox dominates, and N is lost from the system. Critically, the coexistence regime spans a range of NO_2_^−^ fate, from N loss to N retention. Thus, the model indicates that the presence of active denitrification does not necessarily mean that there is more N loss than N retention, and the presence of active DNRA does not mean that there is more N retention than N loss. Next, we examine the resource ratio that allows for an exact balance between N retention and N loss, for anaerobic metabolisms alone, and how this relates to the anaerobic microbial community structure.

### Net zero nitrogen loss

We identify the resource ratio (OM:NO_2_^−^) where NH_4_^+^ produced from DNRA (i.e. retention of inorganic N) balances N_2_ production from denitrification and anammox (i.e. loss of inorganic N), which we refer to as “net zero N loss” (Supplemental Text S3). This balance occurs within the DNRA and denitrification coexistence regime, where anammox is excluded, and where DNRA and denitrification are equally competitive for their shared electron acceptor, thus able to reduce equal amounts of NO_2_^−^ (Fig. 4B). In the model equations, this balance is:


(2) \begin{equation*} \frac{1}{y_{N_{Denit}}}\mu{B}_{Denit}=\frac{1}{y_{n_{DNRA}}}\mu{B}_{DNRA} \end{equation*}

If we assume that both populations have similar steady-state growth rates, *μ*, as they do in the chemostat, which is likely the case in the environment if they are relatively similarly sized cells and thus subject to similar grazing rates, we can then solve for the OM:NO_2_^−^ supply ratio that results in net zero N loss as a function of only the biomass yields for the two populations as:


(3) \begin{equation*} OM:N{O_2^{-}}_{supply}=\frac{1}{2}\left(\frac{y_{N_{Denit}}}{y_{OM_{Denit}}}+\frac{y_{N_{DNRA}}}{y_{OM_{DNRA}}}\right) \end{equation*}

Because the ratios of the yields set the CVs, net zero N loss coincides with the geometric mean of the two CVs (Fig. 4). Along this mean, the biomass of the population carrying out DNRA is only slightly less than the total biomass of the two combined populations carrying out denitrification (NO_2_^−^ ➔ N_2_O and N_2_O ➔ N_2_). Thus, the model suggests that nearly equal amounts of DNRA-associated and denitrification-associated biomass in the environment may indicate net (or near net) zero N loss, for a strictly anoxic environment. With our range of baseline biomass yields (Table 1), this net zero N loss point is between 0.1729–0.1820 mol N (in OM) per mol NO_x_ supplied. Because the C in the OM is what ultimately limits the heterotrophs, this range would vary quantitatively but not qualitatively with the C:N stoichiometry of the OM supplied.

## Discussion

We have provided a broadly applicable framework for understanding the ecological dynamics of anaerobic N-cycling microbial communities in anoxic water columns, both freshwater and marine. Specifically, we demonstrate that by considering the free energy of the underlying half reactions, we are able to simulate the ecological outcomes of the interactions between populations carrying out DNRA versus denitrification that have been theoretically hypothesized and observed and modeled for specific taxa [6, 8, 10]. Our model results are also consistent with the results for the sediment environment of Algar and Vallino [8], although we use an alternative modeling framework and methodology that relies on the outcome of modeled ecological interactions rather than optimization. Therefore, our model is amenable to incorporation into more complex, multidimensional biogeochemical models that typically already include explicit microbial populations (e.g. phytoplankton and zooplankton). If incorporated into multi-dimensional global biogeochemical ocean models, which do not consider DNRA (and perhaps more significantly, its competition with denitrification), the presented framework may provide additional insights into global N-cycling. The model may also offer key insights into both contemporary and future ecosystem function where environmental parameters and community composition data are unavailable (i.e. much of the global ocean and inland waters in historically and chronically understudied regions [38–39]).

Our model links mechanistic microbial ecological dynamics to geochemical quantities that are frequently measured in both freshwater and marine environments. For example, the speciation of accumulated inorganic N is reflective of the dominant functional type (Fig. 3E and F, Supplemental Fig. S5). This suggests broad signatures of microbial metabolism in the accumulation patterns of inorganic N species: denitrification and anammox when NO_x_ accumulates, DNRA when NH_4_^+^ accumulates, and coexistence when neither accumulates. These same patterns have been observed in natural ecosystems such as in oligotrophic OMZs (where OM:NO_2_^−^ supply rates are putatively low, mirroring the left side of the panels of Fig. 4) and the hypolimnion of eutrophic lakes (where OM:NO_2_^−^ supply rate would be expected to be high, mirroring the right side of the panels of Fig. 4). In oligotrophic OMZs, NO_2_^−^ accumulation concomitant with denitrification and/or anammox dominance has been observed [37, 40–41] whereas NH_4_^+^ accumulation and DNRA dominance has been observed in the hypolimnion of stratified lakes [42–43]. Therefore, our model captures broadscale, observed patterns from a first-principles understanding of microbial community function. While we have provided a qualitative comparison of our model with environments with putatively high and low OM: NO_2_^−^ supply rate ratios, a next step in this analysis would be to perform a rigorous meta-analysis of referenced and additional texts to provide clear quantitative comparisons of model output and *in situ* observations. To complement in situ observations of natural environments (where exact supply rate ratios are unknown), the model could be further validated with physical chemostat experiments.

Because functional microbial biomass is resolved explicitly, results provide additional hypotheses that can be tested with more direct observations of microbial communities. For example, the model suggests that DNRA may be a significant component of nitrogen cycling in coastal OMZ. This could be tested through various methods including sequencing *in situ* microbial communities. Furthermore, this model provides an initial steppingstone in the quest to generate new insights from the amassed wealth of sequencing data and associated metadata from diverse aquatic environments. The model mechanistically links microbial ecology to biogeochemical fluxes by relating the relative concentrations of functional type biomasses to nutrient transformation rates. Therefore, the modeled biomasses could be compared to gene-based estimates of microbial population abundances whether by converting functional biomass (the concentration of C- or N-based biomass in the water) to abundance using an estimate of the cell quota (amount of C or N per cell) and well conserved gene copy numbers or other established quantitative frameworks. The bulk N transformation rates in the model (e.g. denitrification) can also be converted to a per cell rate using these conversion factors. While novel computational methods for extracting greater insights from sequencing data are a significant undertaking, microbially explicit biogeochemical modeling brings the aquatic sciences one step closer to being able to one day use gene expression to estimate both rate processes and elemental fluxes [44–45].

A specific question raised by our analysis that could be tested with observational methods is in regard to the outcome of the competition between DNRA and anammox for NO_2_^−^. Anammox is competitively excluded in the NO_2_^−^ limited regime by DNRA in the default model (Figs 3  and 4). This suggests that anammox is only sustainable when NO_2_^−^ does not limit microbial growth, which we speculate is the most likely outcome. However, when anammox was given a lower NO_2_^*^ (and thus higher *y*_N_), it was in principle able to outcompete DNRA (Supplemental Figs S2 and S3), yet its requirement for NH_4_^+^ meant that it relied on NH_4_^+^ supplied from DNRA, and thus anammox coexisted with DNRA in the NO_2_^−^ limited regime. Therefore, anammox was sustainable throughout the model domain and all three functional types were able to coexist within the coexistence domain which contains the net zero N loss point. To determine which of the two model scenarios is more likely, observations could test to see whether anammox strains subsist in NO_2_^−^ limited environments [46], and experiments could probe the competition of anammox (ideally, of low NO_2_^−^ adapted strains) versus heterotrophs for NO_2_^−^. In acknowledging that observations of DNRA/ anammox “coexistence” may be attributed to differences between free-living and particle associated microbial communities [47], size-fractionated studies are of particular importance.

The coexistence of all three functional types (DNRA, anammox, and denitrification) has been observed in natural systems. For example, Kalvelage *et al.* [37] and Roland *et al.* (2018) both measured simultaneous DNRA, anammox, and denitrification in coastal OMZs and Lake Kivu, respectively [48]. The narrow range over which the model was able to simulate coexistence of the three functional types is likely due to the strict competitive exclusion of the simple model. This differs from the environment, where density-dependent mortality, due to “kill-the-winner” grazing [49] or viral lysis, may prevent complete competitive exclusion. Alternatively, anammox could, in principle, co-exist with the other metabolisms when neither OM or N are limiting, as has been demonstrated in instances of coupled and synergistic DNRA and anammox consortia [50–53].

Our model is designed to indicate broad, regionally and globally relevant patterns [54–55], and so we do not expect our model to be able to capture all of the heterogeneity of empirical observations. The current model does not resolve many of the complexities inherent in natural systems, such as niche heterogeneity, complex mortality dynamics (such as viral lysis, which would dampen competitive exclusion), chemoautotrophic and non-canonical DNRA and denitrification [31, 56], and metabolic flexibility which may vary the internal redox constraint for some species [11]. Our framework could be extended to consider some of these additional aspects, such as the chemoautotrophic analogs and mixotrophic denitrification [31, 57] and other NO_2_^−^ reduction pathways including Feammox [58]. The model’s plasticity provides an adaptable tool for understanding the role that DNRA and denitrification competition plays in determining retention and loss of inorganic N across globally distributed and diverse anoxic water columns.

## Conclusion

By basing our model on a fundamental predictor of microbial metabolism (the underlying redox chemistry), we provide a theoretically grounded and computationally rigorous understanding of anaerobic N-cycling microbial communities in anoxic water columns without dependency on site specific data or species-specific traits. This paves a way towards an improved prediction of globally relevant biogeochemical fluxes in unobserved environments (including future conditions). We identified the transition point at which inorganic N retention by DNRA balances inorganic N loss by denitrification (and also anammox), resulting in net zero N loss. The relative loss or retention of bioavailable N is of critical importance to N limited aquatic ecosystems, both inland and marine.

The model provides a universal understanding of the controls on N retention versus loss that also resolves functional microbial biomass. With additional efforts, the model outputs may be converted to and compared with microbial abundances inferred from sequencing data as well as measured per cell activity rates. Therefore, the presented model may contribute to global biogeochemical models as well as provide a tool for hypothesis generation and testing across scales.

## Supplementary Material

SuppMat_Figures_ycaf219

SuppMat_Tables_ycaf219

SuppMat_Text_ycaf219

## Data Availability

Code is archived at https://doi.org/10.5281/zenodo.17635251. All data analyzed in this publication was generated by the available code.
