# Supplementary material for: Redox-constrained microbial ecology dictates nitrogen loss versus retention
Source: ISME Commun. 2025 Nov 25;5(1):ycaf219. doi: 10.1093/ismeco/ycaf219 (PMC12700160; doi:10.1093/ismeco/ycaf219)
Supplement: SuppMat_Figures_ycaf219 [file suppmat_figures_ycaf219.pdf]

Supplemental Materials (Figures) for

Redox-constrained microbial ecology dictates nitrogen loss versus retention

Jemma Fadum, Xin Sun, and Emily Zakem

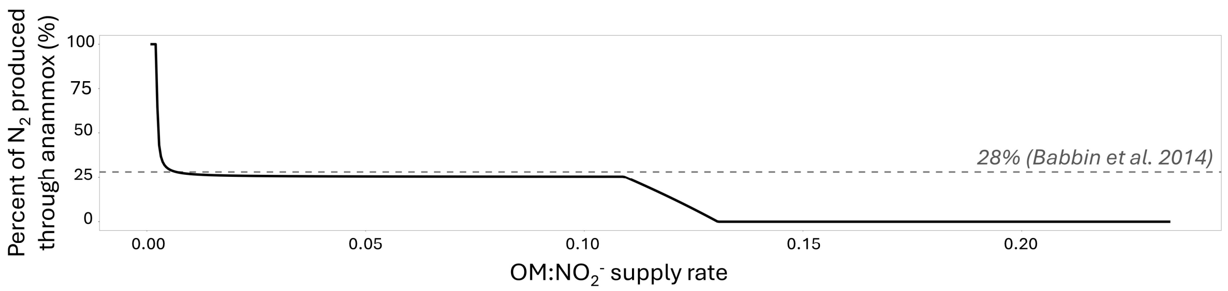

Figure S1. Percentage of N_2 generated in the chemostat by anammox (from both the reduction of NO_2^- and oxidation of NH_4^+).

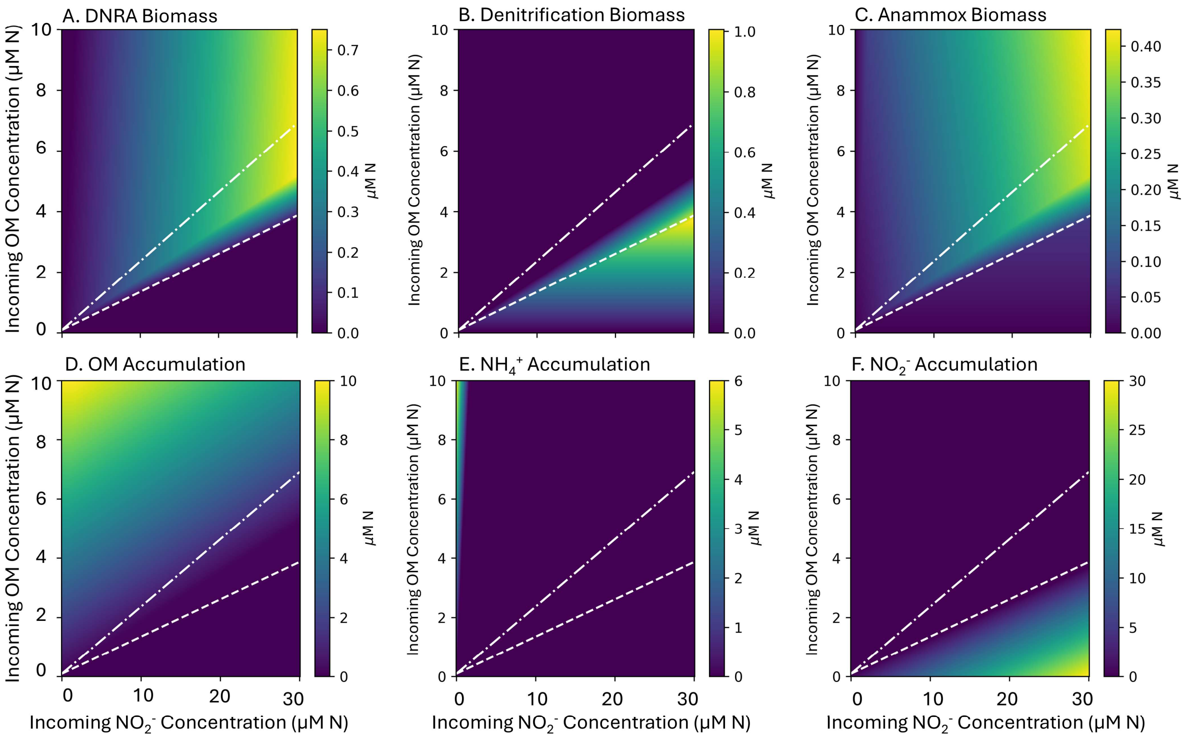

Figure S2. *Increased competitiveness of anammox scenario:* Multiple steady-state solutions of the ecosystem model when anammox has the lowest NO_2* with anaerobic N-cycling metabolic functional types across varying supply of OM and NO_2^-, showing biomass of A) DNRA, B) Denitrification ($NO_2^- \rightarrow N_2O$ + $N_2O \rightarrow N_2$) and C) Anammox. Chemostat accumulation (steady-state concentrations) of D) OM, E) NH_4^+ with F) NO_2^-. The white lines indicate the consumption vectors for DNRA (–•–•–) and denitrification (---). Axis supply rates indicate resource supplies without dilution (0.04).

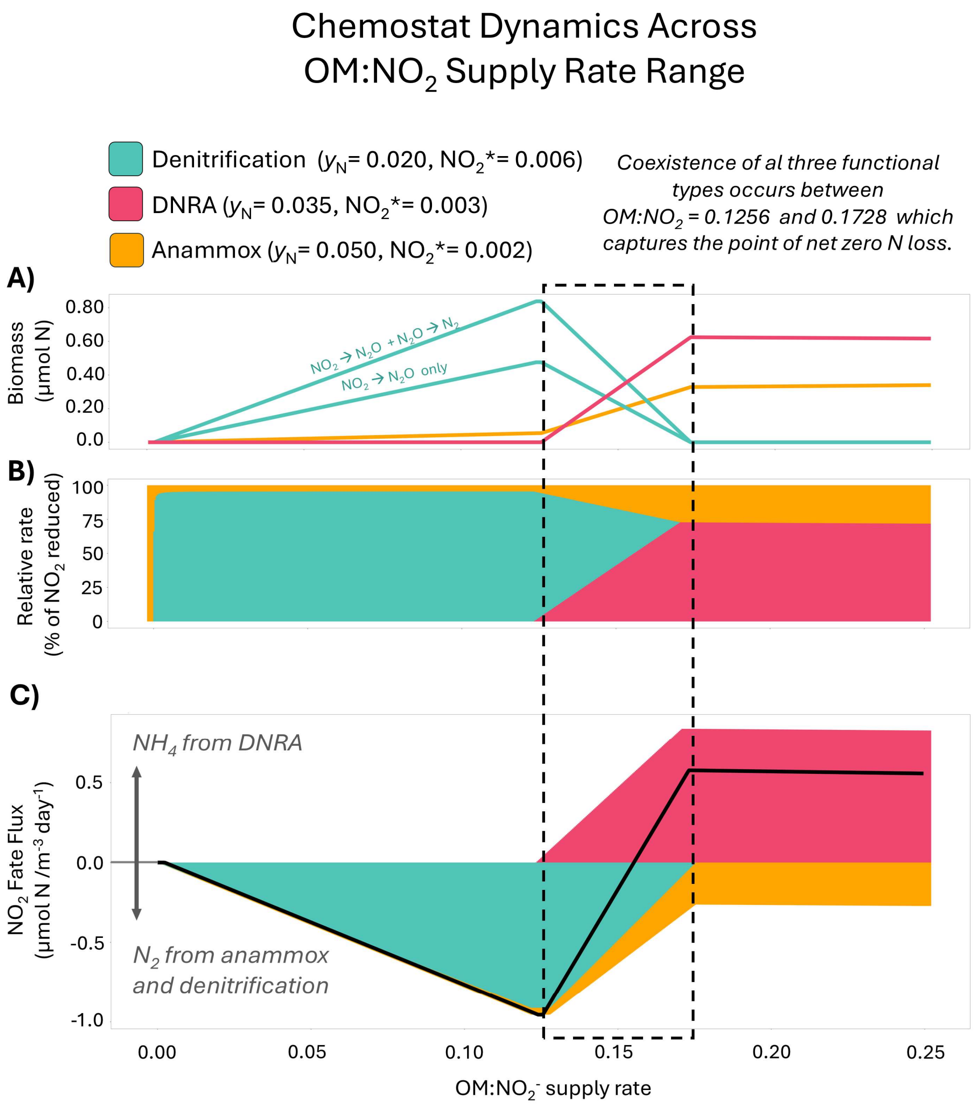

Figure S3. *Increased competitiveness of anammox scenario:* Ecological and biogeochemical outcomes governing the fate of NO_2^- in the ecosystem model when anammox has the lowest NO_2* as a function of the relative supply of OM to NO_2^- (here specifically with incoming NO_2^- concentration of 25 µM N and variable OM supply rate). A) Biomasses of DNRA, denitrification, and anammox functional types, B) Percentage of NO_2^- reduced via the three functional types, C) N_2 and NH_4^+ production with solid black line denoting net balance (different than default model because balance includes N loss from anammox). Range of coexistence for all three functional types contained in dashed line box.

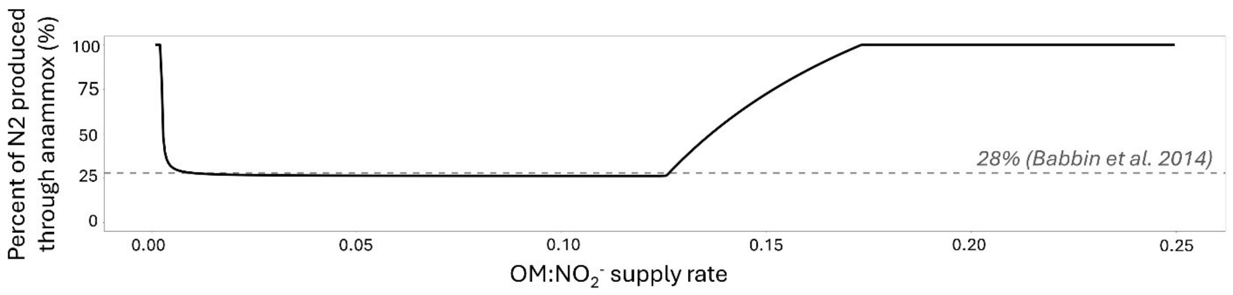

Figure S4. *Increased competitiveness of anammox scenario:* Percentage of N_2 generated in the chemostat by anammox (from both the reduction of NO_2^- and oxidation of NH_4^+) when anammox has the lowest NO_2*.

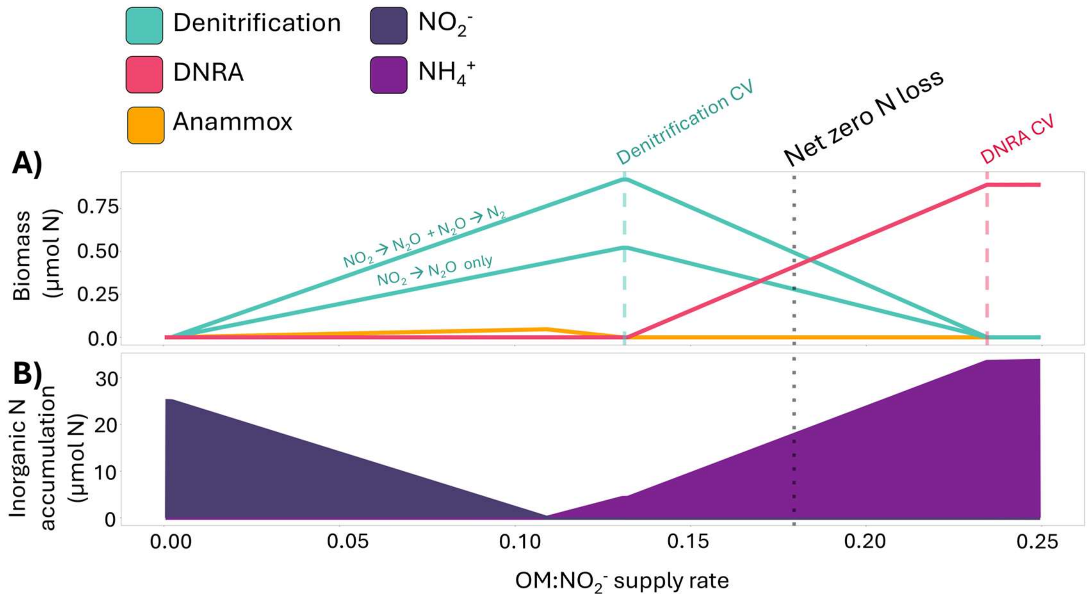

Figure S5. Ecological and biogeochemical outcomes governing the fate of NO_2^- in the ecosystem model (default version) as a function of the relative supply of OM to NO_2^- (here specifically with incoming NO_2^- concentration of 25 μM N and OM supply rate varying). A) Biomasses of DNRA, denitrification, and anammox functional types, same as Figure 4A in main text for reference, B) Speciation of inorganic N accumulation.
