# Supplementary material for: Redox-constrained microbial ecology dictates nitrogen loss versus retention
Source: ISME Commun. 2025 Nov 25;5(1):ycaf219. doi: 10.1093/ismeco/ycaf219 (PMC12700160; doi:10.1093/ismeco/ycaf219)
Supplement: SuppMat_Tables_ycaf219 [file suppmat_tables_ycaf219.pdf]

Supplemental Materials (Tables) for

Redox-constrained microbial ecology dictates nitrogen loss versus retention

Jemma Fadum, Xin Sun, and Emily Zakem

Table S1. Fixed values in model

OM incoming concentration range	0-10 µM N, increments of 0.01 µM N
NO_2^- incoming concentration range	0-30 µM N, increments of 0.1 µM N (Fig. 3); 25 µM N (Fig. 4)
Dilution rate	0.04 /day (Sun et al., 2024)
Simulation total time	2000 days (ensuring equilibrium has been reached)
Timestep for numerical integration	0.001 day

Table S2. Key kinetic parameters for metabolic functional types.

Substrate	Parameter	Value	Source(s)
OM	Maximum uptake rate (V^{max})<br>Half-saturation conc. (K_{OM})	3.0 mols of organic N/ mol N biomass/day<br>1.0 µM	(Jia et al., 2020; Zakem et al., 2020)
NO_2	Maximum uptake rate (V^{max})<br>Half-saturation conc. (K_N)	30 mols of N/ mol N biomass/day<br>0.1 µM	(Awata et al., 2013; Sun et al., 2024)
N_2O	Maximum uptake rate (V^{max})<br>Half-saturation conc. (K_{N2O})	30 mols of N/ mol N biomass/day<br>0.6 µM	(Sun et al., 2017)

References

Anderson, L. A. (1995). On the hydrogen and oxygen content of marine phytoplankton. *Deep Sea Research Part I: Oceanographic Research Papers*, *42*(9), 1675–1680. https://doi.org/10.1016/0967-0637(95)00072-E

Awata, T., Oshiki, M., Kindaichi, T., Ozaki, N., Ohashi, A., & Okabe, S. (2013). Physiological Characterization of an Anaerobic Ammonium-Oxidizing Bacterium Belonging to the "Candidatus Scalindua" Group. *Applied and Environmental Microbiology*, *79*(13), 4145–4148. https://doi.org/10.1128/AEM.00056-13

Jia, M., Winkler, M. K. H., & Volcke, E. I. P. (2020). Elucidating the Competition between Heterotrophic Denitrification and DNRA Using the Resource-Ratio Theory. *Environmental Science & Technology*, *54*(21), 13953–13962. https://doi.org/10.1021/acs.est.0c01776

Rittman, B., & McCarty, P. (2001). *Environmental Biotechnology: Principles and Applications*. McGraw-Hill Education.

Sun, X., Buchanan, P. J., Zhang, I. H., San Roman, M., Babbin, A. R., & Zakem, E. J. (2024). Ecological dynamics explain modular denitrification in the ocean. *Proceedings of the National Academy of Sciences*, *121*(52), e2417421121. https://doi.org/10.1073/pnas.2417421121

Zakem, E. J., Mahadevan, A., Lauderdale, J. M., & Follows, M. J. (2020). Stable aerobic and anaerobic coexistence in anoxic marine zones. *The ISME Journal*, *14*(1), Article 1. https://doi.org/10.1038/s41396-019-0523-8
